# Disease progression in the first 5 years of treatment in multiple sclerosis: Predictive value of early brain and lesion volume changes

**DOI:** 10.1177/13524585231212879

**Published:** 2023-11-29

**Authors:** Rozemarijn M Mattiesing, Eline Kramer, Eva MM Strijbis, Iman Brouwer, Ronald A van Schijndel, Giordano Gentile, Marco Battaglini, Nicola De Stefano, Bernard MJ Uitdehaag, Frederik Barkhof, Hugo Vrenken, Menno M Schoonheim

**Affiliations:** MS Center Amsterdam, Department of Radiology and Nuclear Medicine, Vrije Universiteit Amsterdam, Amsterdam Neuroscience, Amsterdam UMC Location VUmc, Amsterdam, The Netherlands; MS Center Amsterdam, Department of Radiology and Nuclear Medicine, Vrije Universiteit Amsterdam, Amsterdam Neuroscience, Amsterdam UMC Location VUmc, Amsterdam, The Netherlands; MS Center Amsterdam, Department of Neurology, Vrije Universiteit Amsterdam, Amsterdam Neuroscience, Amsterdam UMC Location VUmc, Amsterdam, The Netherlands; MS Center Amsterdam, Department of Radiology and Nuclear Medicine, Vrije Universiteit Amsterdam, Amsterdam Neuroscience, Amsterdam UMC Location VUmc, Amsterdam, The Netherlands; MS Center Amsterdam, Department of Radiology and Nuclear Medicine, Vrije Universiteit Amsterdam, Amsterdam Neuroscience, Amsterdam UMC Location VUmc, Amsterdam, The Netherlands; Department of Medicine, Surgery and Neuroscience, University of Siena, Siena, Italy/SIENA Imaging SRL, Siena, Italy; Department of Medicine, Surgery and Neuroscience, University of Siena, Siena, Italy/SIENA Imaging SRL, Siena, Italy; Department of Medicine, Surgery and Neuroscience, University of Siena, Siena, Italy; MS Center Amsterdam, Department of Neurology, Vrije Universiteit Amsterdam, Amsterdam Neuroscience, Amsterdam UMC Location VUmc, Amsterdam, The Netherlands; MS Center Amsterdam, Department of Radiology and Nuclear Medicine, Vrije Universiteit Amsterdam, Amsterdam Neuroscience, Amsterdam UMC Location VUmc, Amsterdam, The Netherlands; Queen Square Institute of Neurology and Centre for Medical Image Computing, University College London, London, UK; MS Center Amsterdam, Department of Radiology and Nuclear Medicine, Vrije Universiteit Amsterdam, Amsterdam Neuroscience, Amsterdam UMC Location VUmc, Amsterdam, The Netherlands; MS Center Amsterdam, Department of Anatomy and Neurosciences, Vrije Universiteit Amsterdam, Amsterdam Neuroscience, Amsterdam UMC Location VUmc, Amsterdam, The Netherlands

**Keywords:** Brain atrophy, disease progression, early multiple sclerosis, prognostic factors, pseudo-atrophy, white matter lesions

## Abstract

**Background:**

Whether the degree of inflammation (and its resolution) and neurodegeneration after treatment initiation predicts disease progression in multiple sclerosis (MS) remains unclear.

**Objectives:**

To assess the predictive value of magnetic resonance imaging (MRI)-derived brain and lesion volume (LV) changes in years 1 and 2 of treatment for disease progression.

**Methods:**

Patients receiving early interferon beta-1a treatment in REFLEX/REFLEXION (*N* = 262) were included. Predictive regression models included new/enlarging LV (positive activity), disappearing/shrinking LV (negative activity), and global/central atrophy during years 1 and 2.

**Results:**

Faster global atrophy and/or pseudo-atrophy and positive lesion activity in years 1 and 2 related to an increased probability and faster conversion to clinically definite multiple sclerosis (CDMS). Negative lesion activity in year 1 and slower central atrophy in year 2 were predictive of confirmed disability progression (9-Hole Peg Test). Positive lesion activity in year 2 was predictive of faster global atrophy, while positive lesion activity in years 1 and 2 was predictive of faster central atrophy.

**Conclusions:**

A higher degree of global atrophy and/or pseudo-atrophy in year 1 was predictive of CDMS. Positive lesion activity in any year was related to CDMS and neurodegeneration. Disability was related to negative lesion activity in year 1 and slower central atrophy in year 2.

## Introduction

Treatment decisions in multiple sclerosis (MS) are mostly based on the clinical disease course and radiological assessment of new/enlarging T2 lesion activity.^1^ However, MS is also characterized by a neurodegenerative component present from the earliest phases of the disease.^2^

Although previous studies predicting clinical progression mostly focused on the predictive value of lesions,^3^ recent studies pointed out the clinical importance of other pathological mechanisms such as neurodegeneration.^4^ The severity of atrophy can predict disability in MS (independent of lesion metrics)^5^ and is a main candidate for future clinical implementation.^6^ In addition to cross-sectional atrophy, methodological advances now enable longitudinal radiological quantifications of disease progression, such as dynamic changes in lesions and longitudinal global atrophy rates. Regional information, such as ventricular enlargement,^7,8^ can also predict disability progression.

The quantification of “true” neurodegeneration is, however, difficult in settings where treatment is initiated due to pseudo-atrophy. This refers to the rapid resolution of global edema inducing brain volume loss in the first 6 to 12 months after the initiation of anti-inflammatory treatment.^9^ Treatment efficacy is usually assessed by defining breakthrough disease using “positive” lesion activity, that is, new/enlarging T2-hyperintense lesions, T1 gadolinium-enhancing lesions, and spinal cord lesions, all of which have predictive value for disability worsening.^10^ Negative lesion activity (disappearing/shrinking lesions) can reflect the resolution of edema and thus pseudo-atrophy early after the start of treatment. The prognostic potential of both positive and negative lesion activity has not been investigated together. In addition, it remains unclear how to best predict disease progression using a combination of factors during the initial pseudo-atrophy period after treatment initiation and/or the stable treatment period after resolution of edema where neurodegeneration can be quantified more accurately.

In this study, we aimed to investigate which magnetic resonance imaging (MRI) volumetric changes in the first year and second year after treatment initiation are predictive of different measures of clinical progression and neurodegeneration. We used data from the REFLEX/REFLEXION studies, including patients that started early subcutaneous interferon beta-1a treatment, as we have shown the presence of pseudo-atrophy in the first year in this data set.^11^ We now hypothesized that brain and lesion volume (LV) changes in the pseudo-atrophy period have different predictive value for disease progression compared with the period after the resolution of edema.

## Materials and methods

### Data set

REFLEXION (REbif FLEXible dosing in early MS extensION; NCT00813709) was a planned 3-year continuation of the 2-year REFLEX (NCT00404352) study, aimed at assessing the impact of subcutaneous interferon beta-1a treatment on patients with early MS throughout a prolonged period of observation. The REFLEX/REFLEXION studies lasted from 16 November 2006 until 30 August 2013.^12^ Only those patients who started treatment immediately at the beginning of REFLEX (*N* = 262),^11^ the early treatment group, were included in this study. This was chosen due to the study design, as patients in the delayed treatment group started treatment immediately after conversion to clinically definite multiple sclerosis (CDMS), resulting in lowered probabilities of progression in this subgroup over time.

### Measurements

The Expanded Disability Status Scale (EDSS), timed 25-foot walk (T25FW), 9-Hole Peg Test (9HPT), and CDMS were assessed every 3 months during REFLEX and every 6 months during REFLEXION. MRI scans consisting of 1 × 1 × 3 mm^3^ two-dimensional (2D) dual-echo proton density (PD)-/T2-, T1-weighted, and postgadolinium T1-weighted images were acquired yearly within the follow-up period of 5 years.

### Ethical approval

The REFLEX and REFLEXION studies were undertaken in compliance with the Declaration of Helsinki and standards of Good Clinical Practice according to the International Conference on Harmonization of Technical Requirements for Registration of Pharmaceuticals for Human Use. Before initiation of the studies at each center, the relevant institutional review board or independent ethics committee reviewed and approved the study protocols, patient information leaflets, informed consent forms, and investigator brochures. All patients provided written informed consent at the screening visit of REFLEX and before enrollment to REFLEXION.

### Clinical outcome measures

#### CDMS

In REFLEX/REFLEXION,^13,14^ conversion to CDMS was defined as a relapse or sustained progression in EDSS score of ⩾1.5 points compared with baseline and confirmed at the next visit. Time to CDMS conversion was also recorded.

#### Confirmed disability progression

Confirmed disability progression (CDP) was defined by an increase of 1.5, 1, or 0.5 points on the EDSS (for baseline EDSS 0, 1–5, or ⩾5.5, respectively), or ⩾20% worsening on T25FW or 9HPT (in seconds) at any time during the study compared with baseline, all confirmed at the next visit.^15^ EDSS-Plus^16^ progression was also considered, that is CDP on EDSS, T25FW, and/or 9HPT.

### Radiological predictors and outcome measures

Baseline measurements included normalized brain volume (NBV, in L, from SIENAX)^17^, T2-hyperintense lesion volume (T2LV, in mL), and T1 gadolinium-enhancing lesion volume (T1GdLV, in mL). Manual lesion outlines were provided by the Image Analysis Center of Amsterdam UMC (Location VUmc). A semi-automated method^18^ quantified yearly LV changes (in μL). Briefly, PD images of two visits were registered to a halfway space, after slice-to-slice intensity variation and bias-field correction. Then, after intensity matching, images were subtracted and voxel values were converted to Z-scores, based on the mean and standard deviation (SD) within a static “healthy” brain tissue mask. By applying a Z-score threshold inside manual T2 lesion masks (used as the region of interest), the volumes of changing voxels were quantified in four categories: new, enlarging, disappearing, or shrinking; these are reported below as “New LV in year 1,” and so on.

As described in the work by Mattiesing et al.^11^ the yearly percentage brain volume change (PBVC) as a measure of global atrophy and percentage ventricular volume change (PVVC) as a measure of central atrophy were calculated using SIENA^17^ and VIENA,^19^ respectively. A more negative value for PBVC and more positive value for PVVC are indicative of faster atrophy. Neurodegeneration as an outcome measure was based on PBVC and PVVC in the last 3 years of the study, that is, from month 24 to month 60 (PBVC_m24–m60_ and PVVC_m24–m60_, respectively), calculated with formulas provided in the supplemental material.

### Statistical analyses

Statistical analyses were performed with IBM SPSS-28. Stepwise logistic and linear regression were used (manual forward selection, with *p* < 0.05) to identify early imaging features predictive of clinical progression (conversion to CDMS, CDP based on EDSS-Plus, EDSS, T25FW, and 9HPT) and neurodegeneration (PBVC_m24–m60_ and PVVC_m24–m60_), respectively; Cox regression was used for time to CDMS as clinical outcome measure.

**Figure 1.**
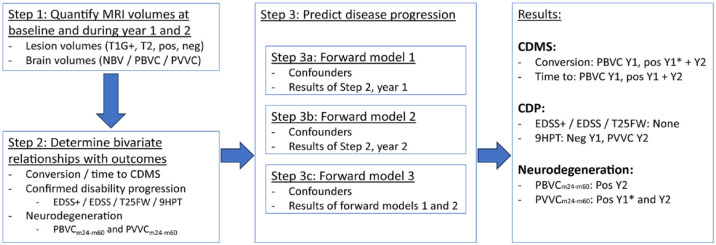
Flowchart depicting the different statistical steps and main results. For more details, see “Materials and methods” section of the main manuscript. 9HPT: 9-Hole Peg Test; CDMS: clinically definite multiple sclerosis; CDP: confirmed disability progression; EDSS: Expanded Disability Status Scale; m24–m60: month 24 to month 60; PBVC: percentage brain volume change; PVVC: percentage ventricular volume change; pos: new and/or enlarging lesions; NBV: normalized brain volume; neg: disappearing and/or shrinking lesions; T1G+: T1 gadolinium-enhancing lesion volume; T2: T2-hyperintense lesion volume; T25FW: timed 25-foot walk; Y1: year 1; Y2: year 2; *did not remain significant in Step 3c.

See Figure 1 for a flowchart depicting subsequent analyses. LV changes and PBVC/PVVC in year 1 and year 2 were first assessed in separate year 1 and year 2 MRI change models as potential individual predictors. Significant predictors from these models were combined in a final model using the “Enter” method. Variables of noninterest were included as confounders for all three models; that is, age, sex, baseline value of the specific clinical outcome measure (EDSS, 9HPT, or T25FW in seconds), and baseline MRI measures (NBV, T2LV, and T1GdLV). PBVC and PVVC year 1 and year 2 were not used as predictors for PVVC_m24–m60_ and PBVC_m24–m60_, respectively.

The following models with potential predictors for all clinical and radiological outcome measures were analyzed:

Model 1. Year 1 MRI change: New LV in year 1, enlarging LV in year 1, disappearing LV in year 1, shrinking LV in year 1, PBVC year 1, and PVVC year 1.
Model 2. Year 2 MRI change: New LV in year 2, enlarging LV in year 2, disappearing LV in year 2, shrinking LV in year 2, PBVC year 2, and PVVC year 2.
Model 3. Combined MRI change: A combination of all significant predictors of models 1 and 2.
All models always included the following variables as confounders: Age, sex, clinical outcome-specific baseline value, baseline NBV, baseline T2LV, and baseline T1GdLV.

### Data availability statement

Any requests for data by qualified scientific and medical researchers for legitimate research purposes will be subject to the Data Sharing Policy of the health care business of Merck KGaA, Darmstadt, Germany. All requests should be submitted in writing to the data sharing portal for the health care business of Merck KGaA, Darmstadt, Germany https://www.emdgroup.com/en/research/our-approach-to-research-and-development/healthcare/clinical-trials/commitment-responsible-data-sharing.html. When the health care business of Merck KGaA has a co-research, co-development, or co-marketing or co-promotion agreement, or when the product has been out-licensed, the responsibility for disclosure might be dependent on the agreement between parties. Under these circumstances, the health care business of Merck KGaA will endeavor to gain agreement to share data in response to requests.

## Results

Table 1 describes the demographics of the cohort (e.g. age (*mean ± SD*) = 31.7 ± 8.4 years, % female = 61.8, and median EDSS = 1.5). The supplemental material shows the individual predictive value of baseline MRI confounders and the bivariate relationships for all outcome measures on which the order of the stepwise forward procedures was based.

**Table 1.** Demographic, clinical, and radiological variables.

Variables	Baseline	
Age (years), mean (SD)	31.676 (8.430)	
Sex (female), *N* (%)	162 (61.832)	
EDSS, median (min, max)	1.5 (0.0, 4.0)	
T25FW (seconds), mean (SD)	6.484 (5.474)	
9HPT (seconds), mean (SD)	20.616 (11.000)	
T1GdLV (mL), median (min, max)	0.000 (0.000, 2.953)^a^	
T2LV (mL), median (min, max)	2.139 (0.020, 25.318)	
NBV (L), mean (SD)	1.531 (0.070)	
Variables	Year 1	Year 2
PBVC (%), mean (SD)	−0.541 (0.722)	−0.353 (0.603)
PVVC (%), mean (SD)	6.868 (7.095)	2.373 (4.582)
NewLV (μL), median (min, max)	19.067 (0.000, 6259.377)	0.000 (0.000, 5430.227)
EnlLV (μL), median (min, max)	22.889 (0.000, 4091.259)	21.458 (0.000, 6784.006)
DisLV (μL), median (min, max)	0.000 (0.000, 703.812)	0.000 (0.000, 151.634)
ShrinkLV (μL), median (min, max)	165.939 (0.000, 10,703.097)	32.902 (0.000, 3298.756)

9HPT: 9-Hole Peg Test; DisLV: disappearing lesion volume; EDSS: Expanded Disability Status Scale; EnlLV: enlarging lesion volume; NBV: normalized brain volume; NewLV: new lesion volume; PBVC: percentage brain volume change; PVVC: percentage ventricular volume change; SD: standard deviation; ShrinkLV: shrinking lesion volume; T1GdLV: T1 gadolinium-enhancing lesion volume; T25FW: timed 25-foot walk; T2LV: T2-hyperintense lesion volume.

a At baseline, 58.397% had no T1 gadolinium-enhancing lesions.

### Clinical outcome: CDMS

A total of 105 (40.076%) of 262 patients converted to CDMS during the study period. Results of the stepwise forward logistic regressions for the models containing the year 1 MRI change (model 1) and year 2 MRI change (model 2) are shown in Table 2. The final model (model 3) with the combined significant MRI change predictors in year 1 (PBVC and new LV) and year 2 (enlarging LV) showed that PBVC year 1 (odds ratio (OR) = 0.568, 95% confidence interval (CI) = [0.368, 0.876], *p* = 0.010) and enlarging LV in year 2 (OR = 1.001, 95% CI = [1.000, 1.002], *p* = 0.007) remained significantly associated with conversion to CDMS, while new LV in year 1 (OR = 1.0008, 95% CI = [0.9998, 1.0018], *p* = 0.116) did not.

**Table 2.** Stepwise forward logistic (clinical outcomes) and linear (neurodegenerative outcomes) regression analyses for the year 1 and year 2 MRI change models

Conversion to CDMS							
Model	Nagelkerke *R*^2^	χ^2^	*p*	Model	Nagelkerke *R*^2^	χ^2^	*p*
Year 1 MRI change	0.104	18.423	0.010*	Year 2 MRI change	0.099	18.701	0.005*
Predictors year 1 MRI change	OR [95% CI]	Wald	*p*	Predictors year 2 MRI change	OR [95% CI]	Wald	*p*
Age	0.968 [0.933, 1.005]	2.903	0.088	Age	0.974 [0.941, 1.009]	2.094	0.148
Sex (female)	0.740 [0.416, 1.315]	1.055	0.304	Sex (female)	0.743 [0.432, 1.278]	1.153	0.283
T1GdLV-BL (mL)	0.788 [0.347, 1.789]	0.324	0.570	T1GdLV-BL (mL)	0.825 [0.379, 1.795]	0.235	0.628
T2LV-BL (mL)	1.008 [0.930, 1.093]	0.040	0.841	T2LV-BL (mL)	0.990 [0.913, 1.074]	0.056	0.813
NBV-BL (L)	0.308 [0.003, 27.288]	0.265	0.607	NBV-BL (L)	0.156 [0.002, 11.494]	0.717	0.397
PBVC-Y1 (%)	0.554 [0.365, 0.841]	7.689	0.006*	EnlLV-Y2 (μL)	1.001 [1.000, 1.002]	8.177	0.004*
NewLV-Y1 (μL)	1.001 [1.000, 1.002]	5.023	0.025*				
CDP: 9HPT							
Model	Nagelkerke *R*^2^	χ^2^	*p*	Model	Nagelkerke *R*^2^	χ^2^	*p*
Year 1 MRI change	0.118	14.734	0.040*	Year 2 MRI change	0.118	14.703	0.040*
Predictors year 1 MRI change	OR [95% CI]	Wald	*p*	Predictors year 2 MRI change	OR [95% CI]	Wald	*p*
Age	0.983 [0.930, 1.039]	0.388	0.533	Age	0.989 [0.938, 1.044]	0.152	0.697
Sex (female)	0.998 [0.413, 2.413]	0.00002	0.996	Sex (female)	0.946 [0.392, 2.284]	0.015	0.901
9HPT-BL (seconds)	0.913 [0.797, 1.047]	1.694	0.193	9HPT-BL (seconds)	0.838 [0.730, 0.962]	6.288	0.012*
T1GdLV-BL (mL)	0.366 [0.067, 1.989]	1.354	0.245	T1GdLV-BL (mL)	0.966 [0.312, 2.987]	0.004	0.952
T2LV-BL (mL)	0.999 [0.884, 1.128]	0.0004	0.983	T2LV-BL (mL)	1.047 [0.924, 1.187]	0.528	0.468
NBV-BL (L)	0.0611 [0.0001, 43.4595]	0.696	0.404	NBV-BL (L)	0.566 [0.001, 376.567]	0.030	0.864
DisLV-Y1 (μL)	1.007 [1.002, 1.012]	8.255	0.004*	PVVC-Y2 (%)	0.871 [0.781, 0.971]	6.216	0.013*
PBVC_m24–m60_							
Model	*R* ^2^	*F*	*p*	Model	*R* ^2^	*F*	*p*
Year 1 MRI change	0.123	4.534	<0.001*	Year 2 MRI change	0.300	9.624	<0.001*
Predictors year 1 MRI change	*B* [95% CI]	*t*	*p*	Predictors year 2 MRI change	*B* [95% CI]	*t*	*p*
Age	−0.009 [−0.028, 0.009]	−1.014	0.312	Age	−0.014 [−0.030, 0.003]	−1.611	0.109
Sex (female)	−0.298 [−0.588, −0.008]	−2.028	0.044*	Sex (female)	−0.280 [−0.546, −0.014]	−2.083	0.039*
T1GdLV-BL (mL)	−0.040 [−0.418, 0.339]	−0.207	0.836	T1GdLV-BL (mL)	0.168 [−0.198, 0.533]	0.906	0.366
T2LV-BL (mL)	−0.052 [−0.091, −0.012]	−2.576	0.011*	T2LV-BL (mL)	−0.042 [−0.079, −0.005]	−2.233	0.027*
NBV-BL (L)	1.873 [−0.419, 4.164]	1.614	0.109	NBV-BL (L)	1.495 [−0.586, 3.577]	1.419	0.158
				NewLV-Y2 (μL)	−0.0004 [−0.0008, −0.0001]	−2.341	0.020*
				EnlLV-Y2 (μL)	−0.0005 [−0.0007, −0.0003]	−4.650	<0.001*
PVVC_m24–m60_							
Model	*R* ^2^	*F*	*p*	Model	*R* ^2^	*F*	*p*
Year 1 MRI change	0.136	4.118	<0.001*	Year 2 MRI change	0.399	14.875	<0.001*
Predictors year 1 MRI change	*B* [95% CI]	*t*	*p*	Predictors year 2 MRI change	*B* [95% CI]	*t*	*p*
Age	0.161 [−0.016, 0.337]	1.800	0.074	Age	0.210 [0.064, 0.355]	2.842	0.005*
Sex (female)	1.953 [−0.810, 4.717]	1.396	0.165	Sex (female)	1.374 [−0.939, 3.688]	1.174	0.242
T1GdLV-BL (mL)	0.349 [−3.291, 3.989]	0.189	0.850	T1GdLV-BL (mL)	−1.502 [−4.680, 1.676]	−0.934	0.352
T2LV-BL (mL)	0.474 [0.098, 0.851]	2.488	0.014*	T2LV-BL (mL)	0.429 [0.106, 0.752]	2.624	0.010*
NBV-BL (L)	−5.584 [−27.298, 16.130]	−0.508	0.612	NBV-BL (L)	−0.070 [−18.188, 18.048]	−0.008	0.994
EnlLV-Y1 (μL)	0.0027 [0.0001, 0.0053]	2.034	0.044*	NewLV-Y2 (μL)	0.006 [0.002, 0.009]	3.470	<0.001*
				EnlLV-Y2 (μL)	0.006 [0.004, 0.007]	6.187	<0.001*

9HPT: 9-Hole Peg Test; BL: baseline; CDMS: clinically definite multiple sclerosis; CDP: confirmed disability progression; CI: confidence interval; DisLV: disappearing lesion volume; EnlLV: enlarging lesion volume; m24–m60: month 24 to month 60; MRI: magnetic resonance imaging; NBV: normalized brain volume; NewLV: new lesion volume; OR: odds ratio; PBVC: percentage brain volume change; PVVC: percentage ventricular volume change; T1GdLV: T1 gadolinium-enhancing lesion volume; T2LV: T2-hyperintense lesion volume; Y1: year 1; Y2: year 2.

ORs and regression coefficients are reported per unit increase for the continuous predictor.

* *p* < 0.05. For significant effects the p-value is printed in bold.

In patients who converted to CDMS during the study period, the mean number of days until conversion was 767.371 ± 486.517. Results for models 1 and 2 are shown in Table 3. The final model showed that new LV in year 1 (hazard ratio (HR) = 1.0007, 95% CI = [1.0003, 1.0011], *p* < 0.001), PBVC year 1 (HR = 0.629, 95% CI = [0.466, 0.849], *p* = 0.002), and new LV in year 2 (HR = 1.0004, 95% CI = [1.0001, 1.0007], *p* = 0.003) remained significantly associated with the time to CDMS, while enlarging LV in year 2 (HR = 1.0001, 95% CI = [0.9999, 1.0003], *p* = 0.276) did not.

**Table 3.** Stepwise forward Cox regression analyses with time to conversion to CDMS as outcome for the year 1 and year 2 MRI change models.

Time to CDMS							
Model		χ^2^	*p*	Model		χ^2^	*p*
Year 1 MRI change		31.899	<0.001*	Year 2 MRI change		20.477	0.005*
Predictors year 1 MRI change	HR [95% CI]	Wald	*p*	Predictors year 2 MRI change	HR [95% CI]	Wald	*p*
Age	0.976 [0.949, 1.003]	2.970	0.085	Age	0.984 [0.959, 1.010]	1.455	0.228
Sex (female)	0.764 [0.499, 1.171]	1.528	0.216	Sex (female)	0.757 [0.500, 1.145]	1.738	0.187
T1GdLV-BL (mL)	0.824 [0.460, 1.475]	0.425	0.515	T1GdLV-BL (mL)	0.872 [0.490, 1.551]	0.218	0.640
T2LV-BL (mL)	1.013 [0.952, 1.077]	0.157	0.692	T2LV-BL (mL)	1.005 [0.944, 1.070]	0.029	0.865
NBV-BL (L)	0.395 [0.012, 13.530]	0.266	0.606	NBV-BL (L)	0.270 [0.009, 8.328]	0.560	0.454
PBVC-Y1 (%)	0.618 [0.461, 0.828]	10.367	0.001*	NewLV-Y2 (μL)	1.0003 [1.0000, 1.0006]	4.298	0.038*
NewLV-Y1 (μL)	1.0007 [1.0003, 1.0010]	14.133	<0.001*	EnlLV-Y2 (μL)	1.0002 [1.0000, 1.0005]	5.002	0.025*

BL: baseline; CDMS: clinically definite multiple sclerosis; CI: confidence interval; EnlLV: enlarging lesion volume; HR: hazard ratio; MRI: magnetic resonance imaging; NBV: normalized brain volume; NewLV: new lesion volume; PBVC: percentage brain volume change; T1GdLV: T1 gadolinium-enhancing lesion volume; T2LV: T2-hyperintense lesion volume; Y1: year 1; Y2: year 2.

HRs are reported per unit increase for the continuous predictor.

* *p* < 0.05. For significant effects the p-value is printed in bold.

### Clinical outcome: CDP

#### EDSS-Plus, EDSS, and T25FW

A total of 115 (43.893%) of 262, 48 (18.321%) of 262, and 72 (27.481%) of 262 patients showed CDP based on the EDSS-Plus, EDSS, and T25FW, respectively. For all three clinical outcome measures, models 1 and 2 were not significant (hence not included in Table 2).

#### 9HPT

A total of 33 (12.595%) of 262 patients showed CDP based on the 9HPT, where models 1 and 2 were significant as shown in Table 2. The final model showed that all predictors in models 1 and 2 remained significant: disappearing LV in year 1 (OR = 1.010, 95% CI = [1.003, 1.016], *p* = 0.003), PVVC year 2 (OR = 0.846, 95% CI = [0.753, 0.950], *p* = 0.005), and baseline 9HPT (OR = 0.830, 95% CI = [0.712, 0.969], *p* = 0.018) were significantly associated with 9HPT-based CDP.

### Neurodegenerative outcomes: global atrophy and central atrophy

Data availability and quality^11^ allowed the quantification of atrophy in 169 patients, that is global atrophy (PBVC_m24–m60_: −1.009 ± 0.967%) and central atrophy (PVVC_m24–m60_: 7.511 ± 9.117%) from month 24 to month 60. Results of the stepwise forward linear regressions for models 1 and 2 are shown in Table 2. For global atrophy, only the cross-sectional model 1 was significant (see Table 2), while longitudinal MRI changes in year 1 did not provide additional information (hence, no model 3 was run). For central atrophy, longitudinal measures did provide additional information and the final model showed that new LV in year 2 (*B* = 0.006, 95% CI = [0.002, 0.009], *p* < 0.001), enlarging LV in year 2 (*B* = 0.006, 95% CI = [0.004, 0.008], *p* < 0.001), age (*B* = 0.184, 95% CI = [0.035, 0.333], *p* = 0.016), and baseline T2LV (*B* = 0.432, 95% CI = [0.109, 0.754], *p* = 0.009) were significantly associated with central atrophy, while enlarging LV in year 1 (*B* = −0.00003, 95% CI = [−0.00239, 0.00233], *p* = 0.982) did not remain significant.

## Discussion

This study investigated the predictive value of early brain and LV changes on clinical disability and neurodegeneration. Faster global atrophy and/or pseudo-atrophy and higher new LV in year 1 (the pseudo-atrophy period), and higher enlarging/new LV in year 2 (after resolution of edema) were related to a more likely, and faster, conversion to CDMS. Global, lower limb, and composite (EDSS-Plus) disability were not predicted by volumetric changes in year 1 or year 2. Worsening hand function was predicted by higher disappearing LV in year 1 and slower ventricular widening in year 2. Neurodegeneration was predicted by earlier positive lesion activity, with some differences between global atrophy and central atrophy.

The predictive value of positive lesion activity for CDMS is evident as observed previously, for instance in another trial on interferon beta.^20^ The observation of the 5-year predictive value of faster global atrophy and/or pseudo-atrophy in year 1 in our study is a novel finding. In early MS patients treated with interferon beta-1a, this could indicate that patients with a stronger immune response have more inflammation and edema. In fact, the severity of pseudo-atrophy directly after treatment initiation in these patients could represent a marker of more aggressive disease and for a higher risk of experiencing a relapse, as the majority of the converted patients in this data set reached CDMS based on the relapse criterion.^14^ For this reason, in this study conversion to CDMS and time to CDMS conversion can be considered a reasonable proxy for the occurrence of a relapse.

For EDSS-Plus, EDSS, and T25FW, atrophy and LV changes in year 1 and year 2 were not predictive in any model. Although imaging predictors were not significant, we found that a worse clinical score at baseline was generally related to a lower probability of disease progression. Although this may sound counterintuitive, this might be explained by regression to the mean.^21^ Alternatively, other factors could be at play, for instance that those who were physically better at baseline might have more to lose than those who started off worse and might have already experienced a deterioration, perhaps due to spinal cord pathology. The probability of progression is then higher for individuals with better baseline scores.

Progression based on hand function was predicted by higher disappearing LV in year 1 and slower central atrophy in year 2. The fact that predictors of 9HPT-based CDP were not relevant for prediction of the other clinical outcome measures might indicate that it is better to look at the separate tests, that is that ambulation and hand dexterity provide complementary information. In addition, this indicates the value of individual measures, instead of a composite (like the EDSS-Plus), since these predictors were not significant for EDSS-Plus-based CDP. Clinically, the importance of individual measures is also clear, as the overlap between 9HPT progressors and T25FW progressors was only 5.3%. The different measures of physical functioning (EDSS, T25FW, and 9HPT) might touch upon different domains and mechanisms in the brain (especially the 9HPT, as the EDSS highly relies on ambulation). Scores on the EDSS and T25FW are associated with spinal cord damage,^22^ which was not assessed in this study, while the 9HPT also touches upon areas that are related to motor planning, information processing, and cognitive pathways in the brain.^23^ Disappearing lesions in year 1 might be interpreted as beneficial, but our results could indicate that a higher degree of resolution of edema could, as discussed above, be a marker of a more aggressive immune response. A recent study also found that shrinking LV was related to worse clinical outcome^24^ but their measurement concerned atrophied LV which is distinct from the negative lesion activity as assessed in this study. The applied semi-automated method is unable to quantify the replacement of lesion tissue by cerebrospinal fluid over time. Another study that also included early MS patients showed that white matter lesion shrinking was not associated with clinical outcome based on the EDSS,^25^ which is in accordance with our findings as we only found a predictive value of disappearing LV for 9HPT-based CDP. A systematic review suggested that T2 lesion counts might have a stronger predictive value than volumes for concurrent EDSS progression.^26^ Effects might become blurred due to the wider variety in volumes, potentially explaining the lack of predictive value for several clinical outcomes in our study. The prognostic value of slower ventricular widening in year 2 for worsening hand function seems counterintuitive, but could potentially indicate that the brain is more slowly recovering from the inflammatory processes of the first year related to global edema in progressors. Alternatively, the amount of brain volume loss in year 1 could have been driven especially by neurodegeneration leading to a floor effect in year 2 in progressors. However, we cannot exclude that this should be considered as a coincidental finding.

Higher positive lesion activity in year 1 was predictive of faster central atrophy and in year 2 of faster global and central atrophy. This is consistent with earlier work showing the predictive value of lesions for subsequent atrophy;^27,28^ however, the novelty of this study lies in predicting atrophy rates while most studies^28^ predicted brain volume at a specific timepoint (cross-sectional atrophy). Previous studies predicting neurodegeneration also focused on gray matter volume or cortical thickness as outcome measures,^29^ which was not feasible in the current data set because of the limited contrast between the white matter–gray matter border in these 2D images. When combining the significant year 1 and year 2 MRI change predictors in the same (final) model, only positive lesion activity in year 2 remained predictive of central atrophy. As such, neurodegeneration seems to have been mainly driven by positive lesion activity in year 2, indicating that resolution of edema (negative lesion activity and pseudo-atrophy) might not be related to eventual neurodegenerative outcomes. This could indicate that the severity of pseudo-atrophy might be related to a worse clinical prognosis (as discussed above), while pseudo-atrophy is not necessarily related to worse neurodegeneration.

### Limitations

Spinal cord lesions were not quantified in this study, although these have been shown to be predictive of disease progression and functioning, which could have driven our lack of predictive power for the EDSS and T25FW.^22,30^ Gray matter atrophy could not be determined on these images, although this has been shown to be more predictive of disease progression than white matter atrophy,^31^ and would also have allowed investigations of the compartmentalization of pseudo-atrophy in these tissue types.^32^ The co-occurrence of pseudo- and actual atrophy in year 1 could have obscured a predictive effect of pseudo-atrophy (except for CDMS) and shrinking lesions. Individual responses to treatment (and treatment adherence) could have introduced more heterogeneity in this data set, as high efficacy therapy was not applied, where the pseudo-atrophy phenomenon might be more pronounced.^33^ In addition, such heterogeneity might also be different at different stages and phenotypes of the disease. It would have been interesting to precisely study the relationship with relapses but this was not feasible with the available data. However, given that in REFLEX only 1 out of 130 patients converted based on EDSS progression,^14^ conversion to CDMS and time to CDMS conversion can be considered a reasonable proxy for the occurrence of a relapse. In future studies, the predictive values of year 1 and year 2 MRI volumetric changes should be investigated with more advanced sequences such as isotropic 3D-FLAIR (three-dimensional fluid-attenuated inversion recovery).

## Conclusion

In the first year of MS, an increased degree of atrophy and/or pseudo-atrophy and disappearing lesions, reflecting resolving inflammation, seem to, respectively, have predictive value for a higher risk of/earlier time to conversion to MS and worsening hand function, while slower ventricular widening in year 2 was predictive of worsening hand function. In both the first year and second year of MS, higher enlarging and new lesion activity were predictive of a higher risk of/earlier time to conversion to MS and faster neurodegeneration across the last 3 years of the study.

## Supplemental Material

sj-pdf-1-msj-10.1177_13524585231212879 – Supplemental material for Disease progression in the first 5 years of treatment in multiple sclerosis: Predictive value of early brain and lesion volume changes Click here for additional data file. Supplemental material, sj-pdf-1-msj-10.1177_13524585231212879 for Disease progression in the first 5 years of treatment in multiple sclerosis: Predictive value of early brain and lesion volume changes by Rozemarijn M Mattiesing, Eline Kramer, Eva MM Strijbis, Iman Brouwer, Ronald A van Schijndel, Giordano Gentile, Marco Battaglini, Nicola De Stefano, Bernard MJ Uitdehaag, Frederik Barkhof, Hugo Vrenken and Menno M Schoonheim in Multiple Sclerosis Journal
