# Supplementary material for: Disease progression in the first 5 years of treatment in multiple sclerosis: Predictive value of early brain and lesion volume changes
Source: Mult Scler. 2023 Nov 29;30(1):44–54. doi: 10.1177/13524585231212879 (PMC10782656; doi:10.1177/13524585231212879)
Supplement: sj-pdf-1-msj-10.1177_13524585231212879 – Supplemental material for Disease progression in the first 5 years of treatment in multiple sclerosis: Predictive value of early brain and lesion volume changes [file sj-pdf-1-msj-10.1177_13524585231212879.pdf]

Supplementary material

The global (percentage brain volume change; PBVC) and central (percentage ventricular volume change; PVVC) atrophy over a longer period, namely from visit month 24 to month 60, was calculated by using the following formulas:

$$\mathrm{PBVC}_{month\ 24\ to\ month\ 60} = \left(\left(1 + \frac{\mathrm{PBVC}_{month\ 24-month\ 36}}{100}\right) \cdot \left(1 + \frac{\mathrm{PBVC}_{month\ 36-month\ 48}}{100}\right) \cdot \left(1 + \frac{\mathrm{PBVC}_{month\ 48-month\ 60}}{100}\right) - 1\right) \cdot 100$$

$$\mathrm{PVVC}_{month\ 24\ to\ month\ 60} = \left(\left(1 + \frac{\mathrm{PVVC}_{month\ 24-month\ 36}}{100}\right) \cdot \left(1 + \frac{\mathrm{PVVC}_{month\ 36-month\ 48}}{100}\right) \cdot \left(1 + \frac{\mathrm{PVVC}_{month\ 48-month\ 60}}{100}\right) - 1\right) \cdot 100$$

Supplementary Table 1. Bivariate relationships between each baseline MRI confounder (rows) and the different outcome measures (columns).

Time to CDMS		
**MRI confounders**	**HR [95% CI]**	**p**
**T1GdLV-BL (mL)**	1.103 [0.701,1.735]	0.672
**T2LV-BL (mL)**	1.020 [0.974,1.069]	0.398
**NBV-BL (L)**	0.179 [0.009,3.769]	0.269
**Conversion to CDMS**		
**MRI confounders**	**OR [95% CI]**	**p**
**T1GdLV-BL (mL)**	1.078 [0.591,1.968]	0.806
**T2LV-BL (mL)**	1.022 [0.959,1.088]	0.505
**NBV-BL (L)**	0.131 [0.003,6.635]	0.310
**CDP: EDSS-Plus**		
**MRI confounders**	**OR [95% CI]**	**p**
**T1GdLV-BL (mL)**	1.370 [0.733,2.559]	0.324
**T2LV-BL (mL)**	1.071 [1.001,1.146]	**0.045***
**NBV-BL (L)**	0.302 [0.006,14.768]	0.546
**CDP: EDSS**		
**MRI confounders**	**OR [95% CI]**	**p**
**T1GdLV-BL (mL)**	1.275 [0.603,2.695]	0.525
**T2LV-BL (mL)**	1.054 [0.978,1.137]	0.167
**NBV-BL (L)**	0.475 [0.003,72.275]	0.771
**CDP: T25FW**		
**MRI confounders**	**OR [95% CI]**	**p**
**T1GdLV-BL (mL)**	1.246 [0.631,2.463]	0.526
**T2LV-BL (mL)**	1.067 [0.998,1.140]	0.058
**NBV-BL (L)**	0.530 [0.007,37.594]	0.770
**CDP: 9HPT**		
**MRI confounders**	**OR [95% CI]**	**p**
**T1GdLV-BL (mL)**	0.899 [0.345,2.348]	0.829
**T2LV-BL (mL)**	1.014 [0.923,1.115]	0.769
**NBV-BL (L)**	0.300 [0.001,89.133]	0.679

PBVC$_{m24-m60}$		
**MRI confounders**	**B [95% CI]**	**p**
**T1GdLV-BL (mL)**	-0.254 [-0.597,0.089]	0.146
**T2LV-BL (mL)**	-0.060 [-0.094,-0.026]	**<0.001***
**NBV-BL (L)**	2.654 [0.412,4.895]	**0.021***
**PVVC$_{m24-m60}$**		
**MRI confounders**	**B [95% CI]**	**p**
**T1GdLV-BL (mL)**	3.376 [0.147,6.605]	**0.041***
**T2LV-BL (mL)**	0.601 [0.280,0.921]	**<0.001***
**NBV-BL (L)**	-12.928 [-34.401,8.546]	0.236

9HPT = 9-Hole Peg Test, BL = baseline, CDMS = clinically definite multiple sclerosis, CDP = confirmed disability progression, CI = confidence interval, EDSS = Expanded Disability Status Scale, m24-m60 = month 24 to month 60, PBVC = percentage brain volume change, PVVC = percentage ventricular volume change, NBV = normalized brain volume, T1GdLV = T1 gadolinium-enhancing lesion volume, T2LV = T2-hyperintense lesion volume, T25FW = Timed 25-Foot Walk, *p<0.05. Odds and hazard ratios and regression coefficients are reported per unit increase for the continuous predictor. All analyses were controlled for age, sex, and clinical outcome specific baseline value.

Supplementary Table 2. Bivariate relationships between the predictors of the year 1 and year 2 MRI change models (rows) and all outcome measures (columns) on which the order of the forward stepwise procedures were based.

**Time to CDMS**					
**Predictors year 1 MRI change**	**HR [95% CI]**	**p**	**Predictors year 2 MRI change**	**HR [95% CI]**	**p**
**PBVC-Y1 (%)**	0.634 [0.475,0.847]	**0.002***	**PBVC-Y2 (%)**	1.198 [0.840,1.707]	0.319
**PVVC-Y1 (%)**	1.018 [0.988,1.048]	0.249	**PVVC-Y2 (%)**	1.034 [0.989,1.082]	0.143
**NewLV-Y1 (µL)**	1.0006 [1.0003,1.0009]	**<0.001***	**NewLV-Y2 (µL)**	1.0004 [1.0002,1.0007]	**0.002***
**EnlLV-Y1 (µL)**	1.0001 [0.9997,1.0004]	0.607	**EnlLV-Y2 (µL)**	1.0003 [1.0001,1.0005]	**0.001***
**DisLV-Y1 (µL)**	1.001 [0.999,1.003]	0.225	**DisLV-Y2 (µL)**	1.007 [0.997,1.017]	0.188
**ShrinkLV-Y1 (µL)**	1.0000 [0.9998,1.0002]	0.939	**ShrinkLV-Y2 (µL)**	1.000 [0.999,1.001]	0.993
**Conversion to CDMS**					
**Predictors year 1 MRI change**	**OR [95% CI]**	**p**	**Predictors year 2 MRI change**	**OR [95% CI]**	**p**
**PBVC-Y1 (%)**	0.581 [0.392,0.860]	**0.007***	**PBVC-Y2 (%)**	1.337 [0.845,2.116]	0.214
**PVVC-Y1 (%)**	1.024 [0.985,1.065]	0.227	**PVVC-Y2 (%)**	1.036 [0.977,1.100]	0.240
**NewLV-Y1 (µL)**	1.001 [1.000,1.002]	**0.040***	**NewLV-Y2 (µL)**	1.001 [1.000,1.002]	**0.032***
**EnlLV-Y1 (µL)**	1.0002 [0.9997,1.0008]	0.444	**EnlLV-Y2 (µL)**	1.001 [1.000,1.002]	**0.004***
**DisLV-Y1 (µL)**	1.001 [0.998,1.004]	0.461	**DisLV-Y2 (µL)**	1.007 [0.991,1.023]	0.377

**ShrinkLV-Y1 (µL)**	1.0000 [0.9997,1.0003]	0.838	**ShrinkLV-Y2 (µL)**	1.000 [0.999,1.001]	0.984
**CDP: EDSS-Plus**					
**Predictors year 1 MRI change**	**OR [95% CI]**	**p**	**Predictors year 2 MRI change**	**OR [95% CI]**	**p**
**PBVC-Y1 (%)**	0.782 [0.532,1.152]	0.214	**PBVC-Y2 (%)**	1.051 [0.665,1.661]	0.831
**PVVC-Y1 (%)**	1.021 [0.979,1.064]	0.336	**PVVC-Y2 (%)**	0.967 [0.910,1.027]	0.272
**NewLV-Y1 (µL)**	1.0001 [0.9995,1.0007]	0.708	**NewLV-Y2 (µL)**	1.0003 [0.9997,1.0009]	0.387
**EnlLV-Y1 (µL)**	1.000 [0.999,1.001]	0.955	**EnlLV-Y2 (µL)**	1.0002 [0.9997,1.0007]	0.347
**DisLV-Y1 (µL)**	1.003 [0.999,1.006]	0.139	**DisLV-Y2 (µL)**	0.997 [0.980,1.014]	0.708
**ShrinkLV-Y1 (µL)**	0.9997 [0.9994,1.0001]	0.111	**ShrinkLV-Y2 (µL)**	1.0004 [0.9995,1.0013]	0.367
**CDP: EDSS**					
**Predictors year 1 MRI change**	**OR [95% CI]**	**p**	**Predictors year 2 MRI change**	**OR [95% CI]**	**p**
**PBVC-Y1 (%)**	0.683 [0.417,1.118]	0.130	**PBVC-Y2 (%)**	1.279 [0.687,2.382]	0.438
**PVVC-Y1 (%)**	1.019 [0.967,1.074]	0.473	**PVVC-Y2 (%)**	1.005 [0.927,1.089]	0.904
**NewLV-Y1 (µL)**	1.0004 [0.9997,1.0011]	0.271	**NewLV-Y2 (µL)**	1.000 [0.999,1.001]	0.784
**EnlLV-Y1 (µL)**	1.0002 [0.9995,1.0009]	0.540	**EnlLV-Y2 (µL)**	1.0004 [1.0000,1.0009]	0.076
**DisLV-Y1 (µL)**	1.002 [0.998,1.006]	0.356	**DisLV-Y2 (µL)**	0.997 [0.972,1.023]	0.825

**ShrinkLV-Y1 (µL)**	0.9998 [0.9994,1.0002]	0.403	**ShrinkLV-Y2 (µL)**	1.000 [0.999,1.001]	0.948
**CDP: T25FW**					
**Predictors year 1 MRI change**	**OR [95% CI]**	**p**	**Predictors year 2 MRI change**	**OR [95% CI]**	**p**
**PBVC-Y1 (%)**	0.663 [0.423,1.038]	0.073	**PBVC-Y2 (%)**	0.929 [0.555,1.553]	0.778
**PVVC-Y1 (%)**	1.037 [0.989,1.087]	0.132	**PVVC-Y2 (%)**	0.984 [0.917,1.055]	0.645
**NewLV-Y1 (µL)**	0.999 [0.998,1.000]	0.157	**NewLV-Y2 (µL)**	1.000 [0.999,1.001]	0.980
**EnlLV-Y1 (µL)**	1.0003 [0.9997,1.0008]	0.395	**EnlLV-Y2 (µL)**	1.0002 [0.9998,1.0007]	0.307
**DisLV-Y1 (µL)**	0.999 [0.995,1.002]	0.459	**DisLV-Y2 (µL)**	0.986 [0.961,1.012]	0.286
**ShrinkLV-Y1 (µL)**	0.9998 [0.9995,1.0002]	0.347	**ShrinkLV-Y2 (µL)**	1.001 [1.000,1.002]	0.088
**CDP: 9HPT**					
**Predictors year 1 MRI change**	**OR [95% CI]**	**p**	**Predictors year 2 MRI change**	**OR [95% CI]**	**p**
**PBVC-Y1 (%)**	0.886 [0.506,1.551]	0.672	**PBVC-Y2 (%)**	1.424 [0.696,2.913]	0.333
**PVVC-Y1 (%)**	1.060 [0.995,1.130]	0.073	**PVVC-Y2 (%)**	0.871 [0.781,0.971]	**0.013***
**NewLV-Y1 (µL)**	1.000 [0.999,1.001]	0.670	**NewLV-Y2 (µL)**	1.0006 [0.9999,1.0012]	0.081
**EnlLV-Y1 (µL)**	1.000 [0.999,1.001]	0.933	**EnlLV-Y2 (µL)**	1.0001 [0.9997,1.0006]	0.543
**DisLV-Y1 (µL)**	1.007 [1.002,1.012]	**0.004***	**DisLV-Y2 (µL)**	1.003 [0.980,1.026]	0.819

**ShrinkLV-Y1 (µL)**	0.9998 [0.9993,1.0003]	0.515	**ShrinkLV-Y2 (µL)**	1.000 [0.999,1.001]	0.945
**PBVC$_{m24-m60}$**					
**Predictors year 1 MRI change**	**B [95% CI]**	**p**	**Predictors year 2 MRI change**	**B [95% CI]**	**p**
**PBVC-Y1 (%)**	0.082 [-0.137,0.301]	0.459	**PBVC-Y2 (%)**	-0.120 [-0.373,0.134]	0.352
**NewLV-Y1 (µL)**	-0.000272 [-0.000548,0.000004]	0.053	**NewLV-Y2 (µL)**	-0.0008 [-0.0011,-0.0004]	**<0.001***
**EnlLV-Y1 (µL)**	-0.0002 [-0.0004,0.0001]	0.228	**EnlLV-Y2 (µL)**	-0.0006 [-0.0008,-0.0004]	**<0.001***
**DisLV-Y1 (µL)**	0.001 [-0.001,0.003]	0.208	**DisLV-Y2 (µL)**	0.001 [-0.007,0.009]	0.894
**ShrinkLV-Y1 (µL)**	-0.00002 [-0.00017,0.00013]	0.786	**ShrinkLV-Y2 (µL)**	-0.0002 [-0.0006,0.0002]	0.410
**PVVC$_{m24-m60}$**					
**Predictors year 1 MRI change**	**B [95% CI]**	**p**	**Predictors year 2 MRI change**	**B [95% CI]**	**p**
**PVVC-Y1 (%)**	0.152 [-0.053,0.357]	0.144	**PVVC-Y2 (%)**	-0.005 [-0.345,0.336]	0.978
**NewLV-Y1 (µL)**	0.001 [-0.001,0.004]	0.334	**NewLV-Y2 (µL)**	0.009 [0.006,0.013]	**<0.001***
**EnlLV-Y1 (µL)**	0.0027 [0.0001,0.0053]	**0.044***	**EnlLV-Y2 (µL)**	0.007 [0.005,0.008]	**<0.001***
**DisLV-Y1 (µL)**	-0.015 [-0.032,0.002]	0.077	**DisLV-Y2 (µL)**	0.011 [-0.064,0.087]	0.771
**ShrinkLV-Y1 (µL)**	-0.00014 [-0.0016,0.0013]	0.843	**ShrinkLV-Y2 (µL)**	0.001 [-0.003,0.005]	0.569

9HPT = 9-Hole Peg Test, CDMS = clinically definite multiple sclerosis, CDP = confirmed disability progression, CI = confidence interval, DisLV = disappearing lesion volume, EDSS = Expanded Disability Status Scale, EnlLV = enlarging lesion volume, m24-m60 = month 24

to month 60, NewLV = new lesion volume, PBVC = percentage brain volume change, PVVC = percentage ventricular volume change, ShrinkLV = shrinking lesion volume, T25FW = Timed 25-Foot Walk, Y1 = year 1, Y2 = year 2, *$p<0.05$. Odds and hazard ratios and regression coefficients are reported per unit increase for the continuous predictor. All analyses were controlled for age, sex, clinical outcome specific baseline value, baseline T2-hyperintense lesion volume, baseline T1 gadolinium-enhancing lesion volume, and baseline normalized brain volume.
